# Early Optimization Stages of *Agave lechuguilla* Bagasse Processing toward Biorefinement: Drying Procedure and Enzymatic Hydrolysis for Flavonoid Extraction

**DOI:** 10.3390/molecules26237292

**Published:** 2021-12-01

**Authors:** Zoé P. Morreeuw, Leopoldo J. Ríos-González, Carmen Salinas-Salazar, Elda M. Melchor-Martínez, Juan A. Ascacio-Valdés, Roberto Parra-Saldívar, Hafiz M. N. Iqbal, Ana G. Reyes

**Affiliations:** 1 Centro de Investigaciones Biológicas del Noroeste (CIBNOR), Instituto Politécnico Nacional 195, Playa Palo Santa Rita Sur, La Paz 23096, Mexico; zpmorreeuw@gmail.com; 2 Departamento de Biotecnología, Facultad de Ciencias Químicas, Universidad Autónoma de Coahuila (UAdeC), Blvd. V. Carranza, Republica Oriente, Saltillo 25280, Mexico; leopoldo.rios@uadec.edu.mx; 3 Tecnologico de Monterrey, School of Engineering and Sciences, Monterrey 64849, Mexico; carmen.salinas@tec.mx (C.S.-S.); elda.melchor@tec.mx (E.M.M.-M.); 4 Bioprocess and Bioproducts Research Group, Food Research Department, Universidad Autónoma de Coahuila (UAdeC), Republica Oriente, Saltillo 25280, Mexico; alberto_ascaciovaldes@uadec.edu.mx; 5 CONACYT-CIBNOR, Instituto Politécnico Nacional 195, Playa Palo Santa Rita Sur, La Paz 23096, Mexico

**Keywords:** agro-industrial waste, biorefinery, added-value natural product, flavonoids, drying process, enzyme-assisted extraction, taguchi method

## Abstract

*Agave lechuguilla* agro-waste is a promising renewable material for biorefining purposes. The procurement of added-value co-products, such as bioactive phytochemicals, is required to improve bioprocesses and promote the bio-based economy of the productive areas of Mexico. In this study, we aimed to evaluate the effect of post-harvest management and enzymatic pretreatment as the first stages of the *A. lechuguilla* valorization process. Four drying methods were compared, and enzymatic hydrolysis was optimized to obtain a flavonoid-enriched extract applying ultrasound-assisted extraction. In both experiments, the total phenolic (TPC) and flavonoid (TFC) contents, HPLC-UV flavonoid profiles, and radical scavenging capacity (DPPH) were considered as response variables. The results demonstrated that light exposure during the drying process particularly affected the flavonoid content, whereas oven-dehydration at 40 °C in the dark preserved the flavonoid diversity and antioxidant functionality of the extracts. Flavonoid glycoside recovery, particularly anthocyanidins, was 1.5–1.4-fold enhanced by enzymatic hydrolysis using the commercial mix Ultraflo© under optimized conditions (pH 4, 40 °C, 180 rpm, and 2.5 h) compared to the unpretreated biomass. The extraction of flavonoids from *A. lechuguilla* bagasse can be carried out using a scalable drying method and enzymatic pretreatment. This study confirmed the potential of this agro-waste as a source of marketable natural products.

## 1. Introduction

A global effort to achieve environmental sustainability and product safety is still challenged in retrieving and valorizing agro-industrial wastes [1,2,3,4,5]. In Mexico, the Tampico fiber industry discharges on the surrounding land over 150,000 tons/year of plant residues leading to environmental and health issues [6,7]. The Tampico fiber is traditionally obtained from *Agave lechuguilla* (Asparagaceae), a native species of northeastern Mexico [8,9], with annual productivity of around 55.98 kg/ha [10]. The harvest and carving of the leaves for their high-quality fibers constitute the primary income for inhabitants of arid and semi-arid rural areas [11].

Since 1996, the fiber recovery from wild *A. lechuguilla* has been considered a sustainable activity due to the corresponding Mexican Normative, which ensures regrowth of the central leaves for further harvests [12,13,14]. However, the exploitation of *A. lechuguilla* leaves results in 15% commercialized fiber and 85% waste [7]. The constant accumulation of the *Agave* bagasse led academics and industrialists to consider this renewable feedstock for biorefining purposes [15,16,17]. Due to its large availability and its lack of competition with human foods and animal feeds, *A. lechuguilla* biomass is considered a promising option for bioenergy production [7]. 

Therefore, bioprocesses have already been consolidated [18,19,20,21]. Although, for the economic feasibility of the process, other derived products are required to improve the biorefinery scheme [7,8,9,10,11,12,13,14,15] and establish the commercial value of *A. lechuguilla* bagasse. Following the global trends in the valorization of agro-waste, phytochemical recovery recently appeared as a relevant new step in biorefinery development [22,23,24,25]. Thus, the current proposal for *A. lechuguilla* and other agave-related industries is to obtain high-added-value co-products [15,26]. 

In addition, plants remain the worldwide largest source of natural bioactive products [27], and there is a rising interest in using agro-residues, such as corn husks, as low-cost feedstocks for the procurement of active biomolecules, such as flavonoids [2,28,29]. In this context, bioprospecting studies have already described the benefits of *A. lechuguilla* extracts as antioxidant [30,31], anticancer [32], feed additive [33], antiparasitic [34,35], antibacterial, and antifungal [31,36,37]. Among the active agents of *A. lechuguilla* waste biomass, phenolic compounds have been widely characterized [38,39,40,41]. The diversity of chemical structures of the flavonoids found in *A. lechuguilla* bagasse explain the wide range of exhibited bioactivities. 

The major group of flavonoids found in *A. lechuguilla* co-products are glycoside flavonols with concentrations ranging from 291.51 ± 15.017 to 1251.96 ± 63.09 µg/g Dry Weight (DW) and are known for acting as antioxidant, antibacterial, antiviral, cardio protective, anti-inflammatory, and anti-cancer [32,42]. The second-most abundant compounds are the anthocyanins with about 12.32 to 24.23 µg/g DW and are particularly interesting for their health-promoting, antibacterial, and antioxidant capacities [43,44]. 

At lower concentrations, the aglycone flavonols and flavanols presented, respectively, 15.57 and 7.91 µg/g DW, and showed antioxidant, anti-inflammatory, and anti-cancer effects [45]. The stated concentrations [40,41] and potential therapeutic effects suggest the downstream uses of the *A. lechuguilla* derivative products in cosmetic, nutraceutical, and pharmaceutical industries. In addition, the conservation of the flavonoid profiles through the productive areas confirmed the potential of this abundant plant material for the procurement of natural bioactive ingredients for commercial applications [41].

However, the valorization of agro-waste through the procurement of active phytochemicals requires adequate management and pretreatment of the biomass [25,46]. Morreeuw et al. [40] demonstrated the preservation of flavonoids in the *A. lechuguilla* bagasse stored for nine months under suitable conditions, e.g., preventing light, moisture, and oxygen exposure. Freeze-drying is the best laboratory procedure for phytochemical preservation; however, it is time- and energy-consuming and, thus, unsuitable at an industrial scale. Alternative drying methods have been considered for the use of *Agave* spp. biomass at a larger scale, such as drying under natural conditions (environment temperature and light) [47] and artificial conditions performing oven dehydration at 45 °C [18], 60 °C [47], and 105 °C [48]. 

Temperature and light exposure are the most important factors that might alter the stability of flavonoids structure and function [49,50,51,52]. Hence, if the first insights in the processing of *A. lechuguilla* bagasse for flavonoid extraction suggested large-scale valorization potential, the drying procedure has yet to be adjusted to reach an industrial scale. After that, the plant cell matrix is known for to be a limit in the extraction of phytochemicals, mainly phenolic compounds, linked to the cell wall multilayer structure [53,54]. Acid, alkaline, and thermal pretreatments of *A. lechuguilla* biomass have been studied as previous steps for biogas and biofuel production [18,20,21]. 

However, such processes are known to modulate the physico-chemical properties of the phytochemicals [55,56]. For instance, Carmona et al. [30] reported that alkaline pretreatment decreased the antioxidant capacity of the *A. lechuguilla* extracts. In contrast, enzymatic hydrolysis effectively degrades and disrupts the lignocellulosic matrix to release bioactive flavonoids without affecting their biological properties [57,58]. Thus, the integration of a hydrolysis step prior to extraction could enhance flavonoid recovery. However, critical parameters must be considered to adequately perform enzymatic-assisted extraction of bioactive compounds, such as the enzyme loading, solids loading, pH, temperature, and incubation time [54,59].

In this regard, the present work aimed to optimize the early stages of the bioprocessing of the *A. lechuguilla* agro-waste by targeting highly valuable flavonoids. Different drying methods were applied to determine the impact of light and temperature on the conservation of flavonoid content. Afterward, optimization of the enzymatic pretreatment was conducted through Taguchi-based methods to evaluate the impact of mixed composition and incubation parameters (pH, temperature, and time) in the physicochemical properties of the extracts. In all experiments, the extraction yield, total phenolic content (TPC), total flavonoid content (TFC), quantitative HPLC-UV profile, and free-radical scavenging capacity (DPPH) were assessed. Optimization of the drying and enzymatic pretreatment for the bioconversion of the *A. lechuguilla* bagasse into added-value extracts provides new insights that could be considered in the biorefinery conceptualization and scaling-up approach to reach industrial applications. 

## 2. Results

### 2.1. Drying Process

#### 2.1.1. Extraction Yields

**Figure 1:**
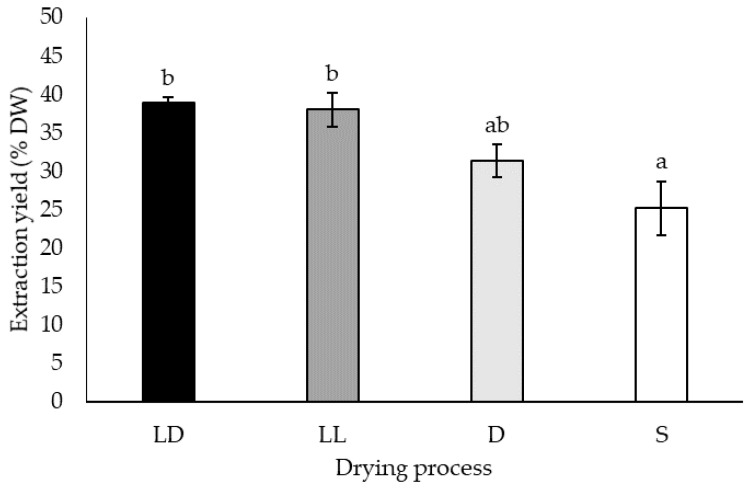
Global extraction yields in percentage of dry weight (% DW) obtained for ethanolic extraction of *Agave lechuguilla* bagasse freeze-dried in the dark (LD), exposed to light (LL), oven-dehydrated (D), and sun-dried (S). The given letters “a”, “b”, and “ab” indicate statistically significant results as per Kruskal–Wallis test (n = 3, *p* < 0.05).

Regarding the drying process, the highest yields were obtained using freeze-drying independently from light exposure, with 38.98 ± 0.64 %DW in the dark (LD) and 38.03% ± 2.18 %DW with light (LL). The oven-dehydration (D) and sun-dry (S) procedures markedly reduced the extraction yields compared to the lyophilized samples, resulting in 31.37 ± 2.17 %DW and 25.16 ± 3.53 %DW, respectively (Figure 1).

#### 2.1.2. Total Phenolic and Flavonoid Content

**Figure 2:**
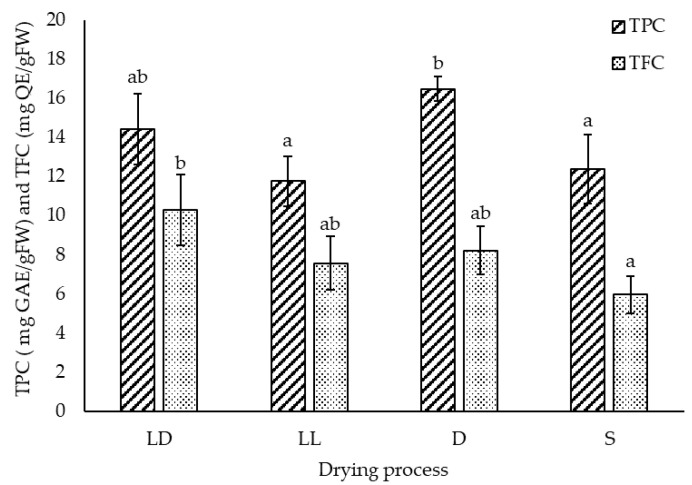
The total polyphenol content (TPC) expressed in milligram gallic acid equivalents (mg GAE) and total flavonoid content (TFC) in milligram quercetin equivalents (mg QE) per gram of fresh weight (FW) measured in ethanolic extracts of *Agave lechuguilla* bagasse freeze-dried in the dark (LD), exposed to light (LL), oven-dehydrated (D), and sun-dried (S). The given letters “a”, “b”, and “ab” indicate significant differences as per Tukey HSD analysis for TPC and TFC data, respectively (n = 12, *p* < 0.05).

The drying process significantly impacted both the phenolic and flavonoid concentrations in the extracts (Figure 2). The highest TPC was obtained from oven-dry bagasse with 16.47 ± 0.63 mg GAE/g Fresh Weight (FW); however, it was not significantly higher than the 14.41 ± 1.81 mg GAE/g FW obtained from the bagasse freeze-dried in the dark. Likewise, the LD (14.41 ± 1.81 mg GAE/g FW), LL (11.75 ± 1.27 mg GAE/g FW), and S (12.38 ± 1.76 mg GAE/g FW) treatments did not show a significant difference in the TPC. Regarding the flavonoid content, TFC was significantly higher in extracts obtained from LD treated bagasse (10.29 ± 1.80 mg QE/g FW) compared to the sun-dried material (5.96 ± 0.96 mg QE/g FW), whereas the TFC obtained from LL and D conditioned biomass, which did not differ from the other drying processes, gave 7.57 ± 1.38 and 8.23 ± 1.22 mg QE/g FW, respectively.

#### 2.1.3. Specific Flavonoid Profiles

The number of flavonoids quantified by HPLC-UV analysis represented approximately 10.64% of the total flavonoid content. The specific recovery yields were 7.66% glycoside flavonols, 2.72% glycoside flavanones, 0.33% aglycone flavonols, 0.09% anthocyanins, and 0.037% aglycone flavanols (Table 1; Figure 2).

The drying process revealed differential abundances between light conditions with the lowest concentrations of all quantified flavonoids for LL and S, and the highest concentrations for LD and D (Table 1). More specifically, isorhamnetin was almost twice as concentrated for oven-dried (864.35 ± 7.70 µg/g DW) as for sun-dried bagasse (458.96 ± 2.04 µg/g DW). Light also impacted the anthocyanin (cyanidin and delphinidin) abundance, which fell under the detection limits for the extracts obtained from bagasse exposed to light. 

Flavanones showed a similar behavior, although no significance was evident between dark conditions with 224.78 ± 9.79 µg/g DW (LD) and 194.12 ± 5.18 µg/g DW (D), and LL-dried biomass (179.29 ± 1.37 µg/g DW). Likewise, quercetin and kaempferol presented comparable LD, LL, and D treatment concentrations with respective means of about 13.21 and 12.97 µg/g DW. In contrast, catechin, naringenin, and apigenin concentrations in the extracts were not affected and remained at around 3.06, 1.43, and 8.14 µg/g DW, respectively.

#### 2.1.4. Free-Radical Scavenging Capacity

**Figure 3:**
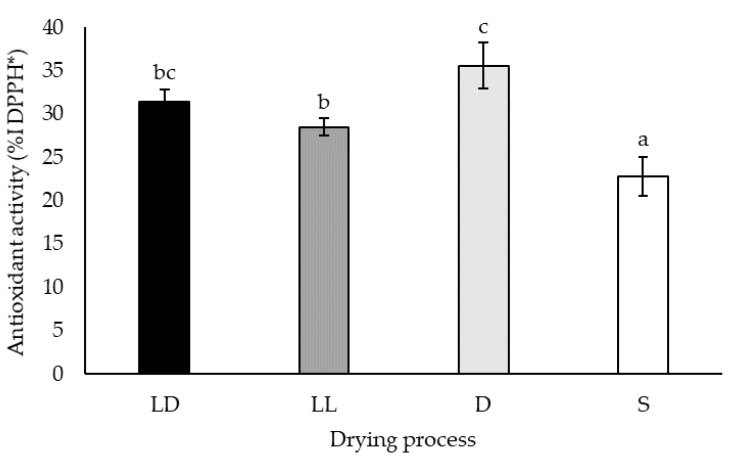
Antioxidant capacity of the ethanolic extracts of *Agave lechuguilla* bagasse freeze-dried in the dark (LD), exposed to light (LL), oven-dehydrated (D), and sun-dried (S) expressed as a percentage of the scavenging of the DPPH radicals (% I DPPH). The given letters “a”, “b”, “c”, and “bc” indicate significant differences as per Tukey HSD analysis (n = 4, *p* < 0.05).

The effect of the drying process on the physicochemical properties of *A. lechuguilla* bagasse extracts was reflected in their free-radical scavenging capacity (Figure 3). The results showed a higher antioxidant activity (AA) obtained with the oven-dried material (35.59 ± 2.68 %I), followed by freeze-dried samples (31.45 ± 1.41 %I, under dark conditions, and 28.54 ± 1.02 %I, under light conditions), and the lowest AA was observed for the sun-dried biomass (22.87 ± 2.25 %I).

### 2.2. Optimization of the Enzymatic Hydrolysis Process

#### 2.2.1. Screening of Enzyme Concentrations

**Figure A1:**
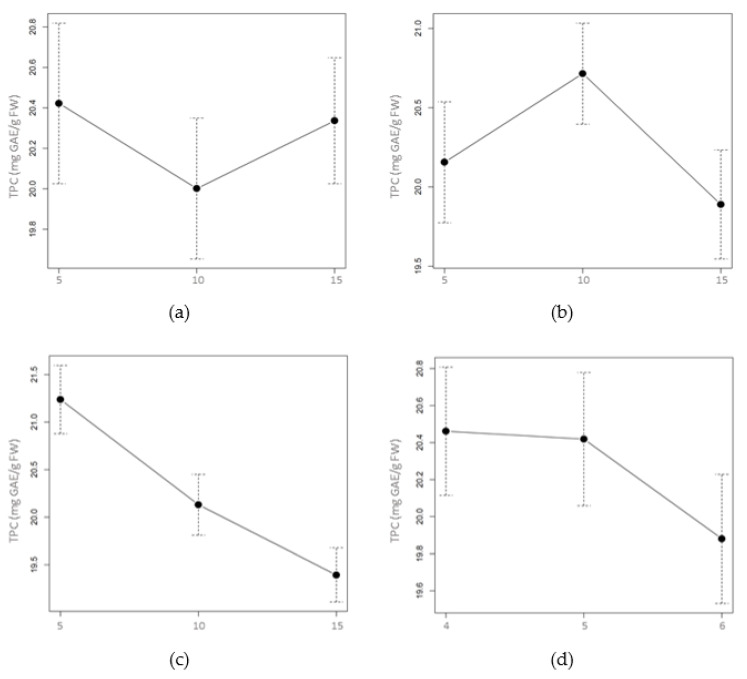
The total phenolic content (TPC; mg GAE/g FW) in the ethanolic extracts plotted as a function of the individual enzyme concentration (5–15 IU), (**a**) cellulase, (**b**) pectinase, (**c**) laccase, and (**d**) pH (4–6).

**Figure A2:**
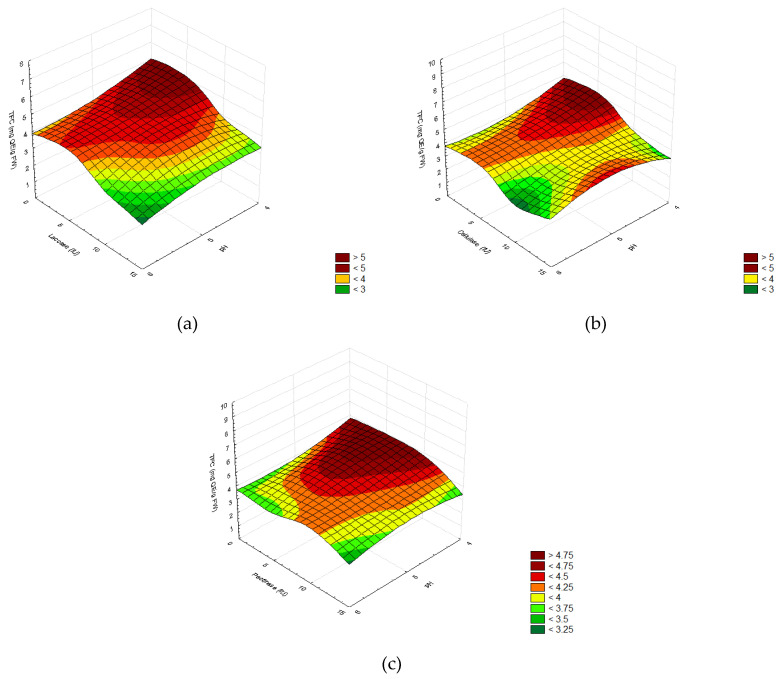
The total flavonoid content (TFC; mg QE/g FW) in the ethanolic extracts plotted as a function of the pH (4–6) and individual enzyme concentration (5–15 IU), (**a**) cellulase, (**b**) pectinase, (**c**) laccase. The color scale indicates significance of the analysis of variance for the Taguchi method (n = 3, *p* > 0.05).

The first experimental design (DOE I) was carried out to screen the effect of the concentrations (5–15 IU) of laccase, cellulase, and pectinase as a function of the pH (4–6) on the extraction yields, TPC, and TFC of the *A. lechuguilla* biomass. The results merged in Table A4 showed that enzymes increased the extraction yields (23.99 ± 2.17 %FW) compared to the controls (18.58 ± 0.22 %FW), and the enzyme concentration did not induce a variation of the extraction yields. Similar trends were observed regarding the total phenolic and total flavonoid contents (Figure A1 and Figure A2). Laccase drastically decreased the TPC and TFC, whereas cellulase and pectinase did not have a significant effect. Modeling of the response variables according to the Taguchi design of the experiment predicted that increasing pH and laccase concentration negatively impacted TPC, an intermediate value of pectinase increased the TPC, whereas cellulase concentration had no effect.

For the following DOE, three cocktails were formulated based on the results of DOE I with laccase, cellulase, and pectinase at respective proportions: 1:1:1 (LCP), 1:1:2 (LCPP), and 0:1:2 (CPP).

#### 2.2.2. Extraction Yields

**Figure 4:**
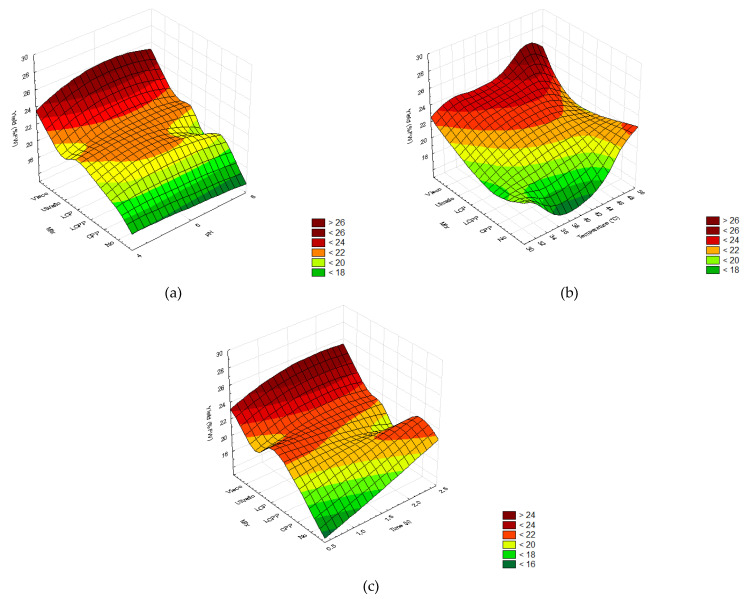
Extraction yields (%FW) plotted as a function of the different enzymatic mixes. (**a**) pH (4–6), (**b**) temperature (30–50 °C), and (**c**) time (0.5–2.5 h). In order, two commercial cocktails: Viscozyme© (Visco) and Ultraflo© (Ultraflo); three formulated cocktails with laccase, cellulase, and pectinase at proportions of 1:1:1 (LCP), 1:1:2 (LCPP), and 0:1:2 (CPP); and the control without added enzymes (No). The color scale indicates the significance of the analysis of variance for the Taguchi method (n = 3, *p* > 0.05).

The extraction yields obtained from DOE II and III were merged to compare the formulated mixtures with laccase, cellulase, and pectinase, the enzymatic cocktails from Novozymes^®^, and the control treatment without enzyme. Globally, the yields remained in the same range as for DOE I with respective means of 20.79 ± 1.76 %FW (DOE II) and 21.23 ± 1.70 %FW (DOE III) (Table A4) and were higher than the control (Figure 4). The best extraction yields were obtained using commercial mix Viscozyme©. In addition, increasing the pH, temperature, and time further enhanced the extraction yields (Figure 4). 

#### 2.2.3. Total Phenolic and Flavonoid Content

A global increase of the TPC and TFC was observed with the treatments applied in DOE II and III compared to the control and results from DOE I. For DOE II, the TPC reached 12.17 ± 0.60 mg GAE/g FW, and TFC reached 3.55 ± 0.56 mg QE/g FW, which were lower than those obtained in DOE III using commercial enzyme mixtures, which yielded TPC of about 14.07 ± 0.2.76 mg GAE/g FW and TFC of 4.89 ± 0.56 mg QE/g FW. However, multifactorial analysis of variance revealed that significance was found only for specific values of the tested factors (Table A4).

**Figure 5:**
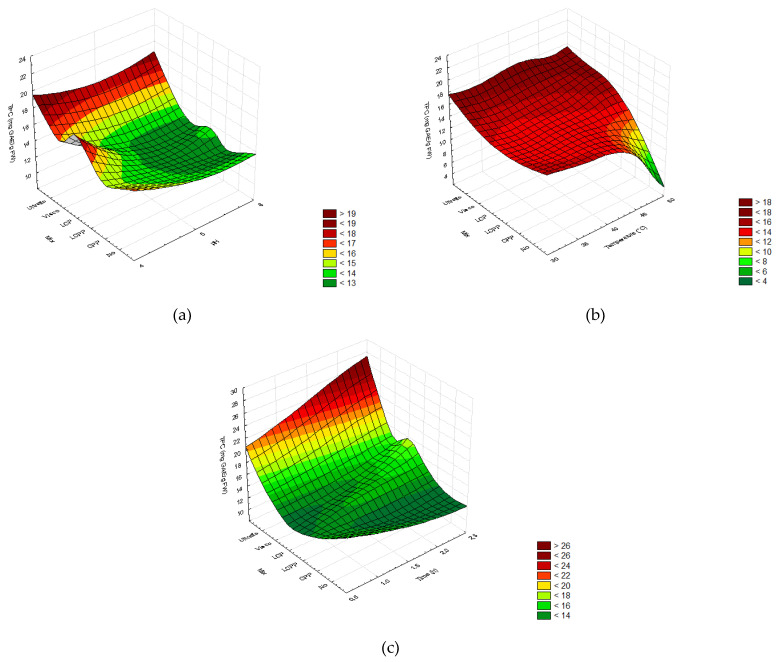
The total phenolic content (mg GAE/g FW) in the ethanolic extracts plotted as a function of the different enzymatic cocktails and; (**a**) pH (4–6), (**b**) temperature (30–50 °C), and (**c**) time (0.5–2.5 h). In order; two commercial cocktails: Ultraflo© (Ultraflo) and Viscozyme© (Visco); three formulated cocktails with laccase, cellulase, and pectinase in proportion 1:1:1 (LCP), 1:1:2 (LCPP), and 0:1:2 (CPP); and the control without enzymes added (No). The color scale indicates the significance of the analysis of variance for the Taguchi method (n = 3, *p* > 0.05).

**Figure 6:**
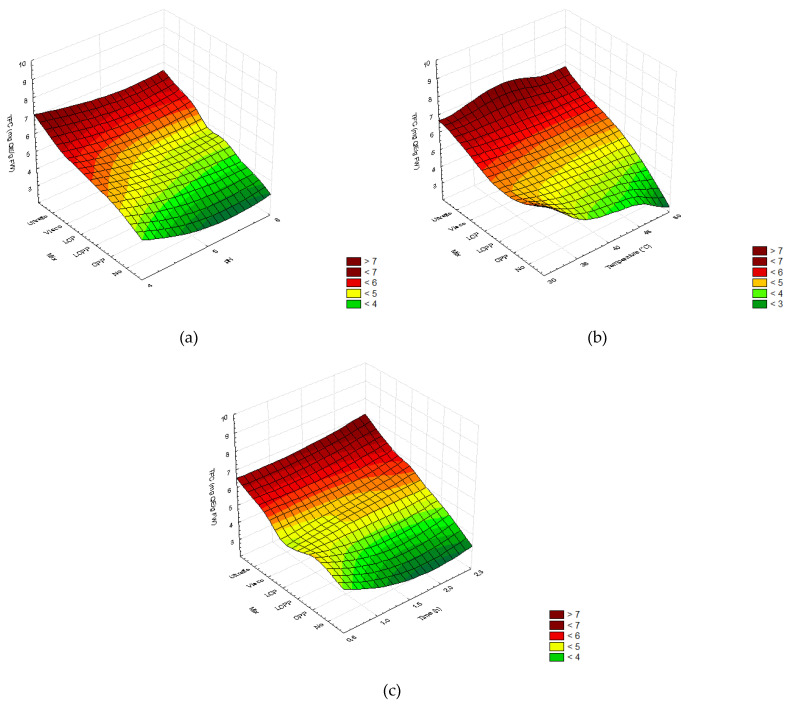
The total flavonoid content (TFC, mg QE/g FW) in ethanolic extracts plotted as a function of the different enzymatic cocktails. (**a**) pH (4–6), (**b**) temperature (30–50 °C), and (**c**) time (0.5–2.5 h). In order, two commercial cocktails: Ultraflo© (Ultraflo) and Viscozyme© (Visco); three formulated cocktails with laccase, cellulase, and pectinase at proportions of 1:1:1 (LCP), 1:1:2 (LCPP), and 0:1:2 (CPP); and the control without added enzymes (No). The color scale indicates the significance of the analysis of variance for the Taguchi method (n = 3, *p* > 0.05).

**Figure A3:**
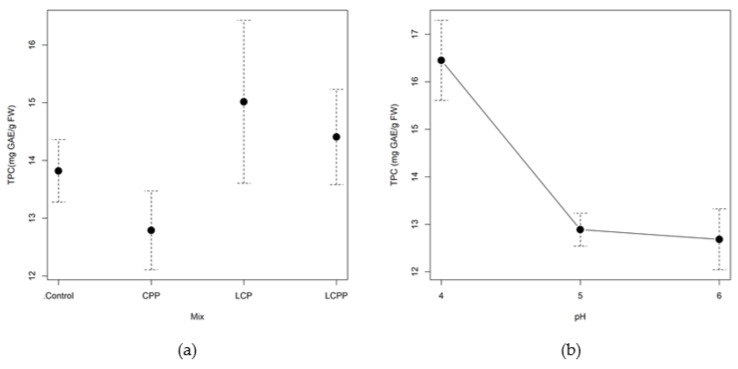

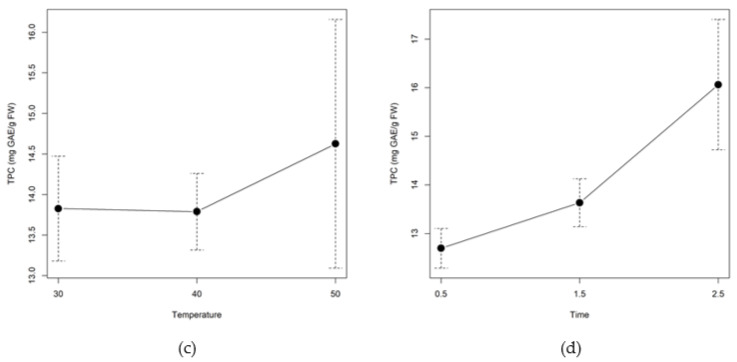
The total phenolic content (TPC; mg GAE/g FW) in ethanolic extracts plotted as a function of the (**a**) enzymatic mix with laccase, cellulose, and pectinase at proportions of 1:1:1 (LCP), 1:1:2 (LCPP), and 0:1:2 (CPP), (**b**) pH (4–6), (**c**) temperature (30–50 °C), and d) time (0.5–2.5 h).

**Figure A4:**
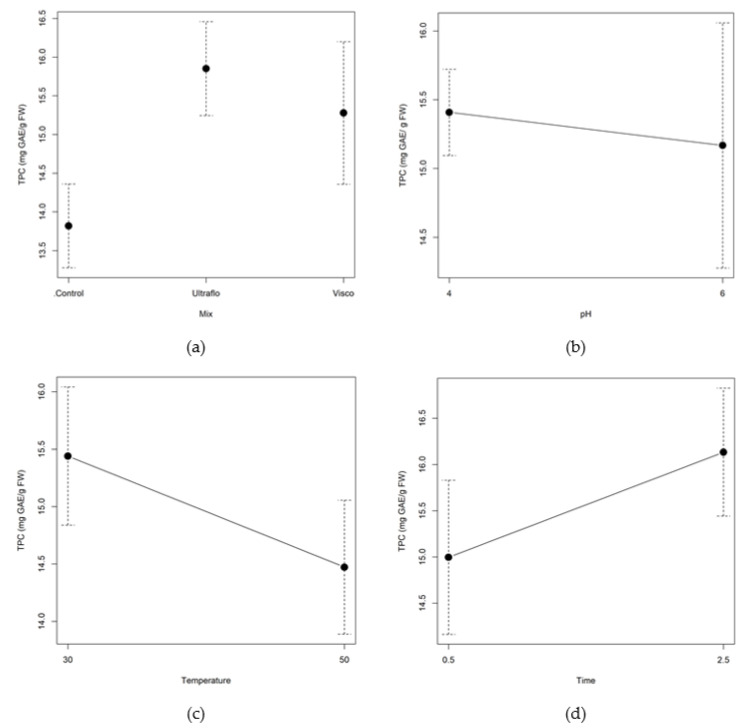
The total phenolic content (TPC; mg GAE/g FW) in ethanolic extracts plotted as a function of the (**a**) enzymatic mix (Ultraflo© and Viscozyme©), (**b**) pH (4–6), (**c**) temperature (30–50 °C) and (**d**) time (0.5–2.5 h).

The highest TPC and TFC were obtained with the enzyme LCP mix: TPC 20.56 ± 2.87 mg GAE/g FW; TFC 5.83 ± 0.83 mg QE/g FW; and the two commercial cocktails, Viscozyme©: TPC, 18.63 ± 3.81 mg GAE/g FW; TFC, 7.38 ±1.50 mg QE/g FW; and Ultraflo©: TPC, 17.13 ± 3.83 mg GAE/g FW, TFC, 6.62 ± 1.48 mg QE/g FW. The TPC was even higher with the lowest pH value (4), intermediate temperature (40 °C), and highest time (2.5 h) (Figure 5 and Figure A3). In contrast, the pH, temperature, and time did not induce TFC variation among individual enzymatic treatments (Figure 6 and Figure A4).

**Figure 7:**
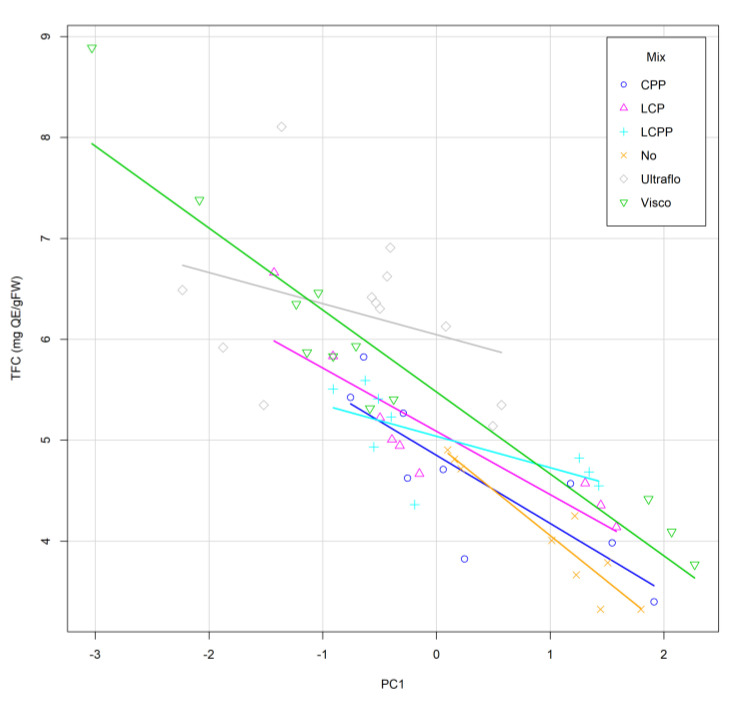
The total flavonoid content (mg QE/g FW) variability explained the 93.79% by the first principal component, PC1, related to the enzymatic cocktail (78.31%) and other factors level (21.69%). Lines represent linear regression of data dispersion within each enzymatic treatment. In order, two commercial cocktails: Ultraflo© (Ultraflo) and Viscozyme© (Visco); three formulated cocktails with laccase, cellulase, and pectinase at proportions of 1:1:1 (LCP), 1:1:2 (LCPP), and 0:1:2 (CPP); and the control without added enzymes (No).

In addition to the modeling based on Taguchi methods, principal component analysis (PCA) was performed in the merged data to classify each tested factor’s effect on the variation of TPC and TFC. The PCA confirmed that variability of TPC is mainly explained by pH (35.18%), enzyme mix (28.57%), incubation time (21.31%), and temperature (14.95%). In contrast, for TFC, the enzymatic cocktail is the main impacting factor (PC1, 78.31%), whereas no significant variation was induced by the pH, temperature, and incubation time and accounted for 21.69% (PC2) of the TFC variability. The dispersion of the TFC plotted according to the PC1 showed that the highest values with the lowest variability were related to the use of Ultraflo© (Figure 7). 

#### 2.2.4. Free-Radical Scavenging Capacity

**Figure 8:**
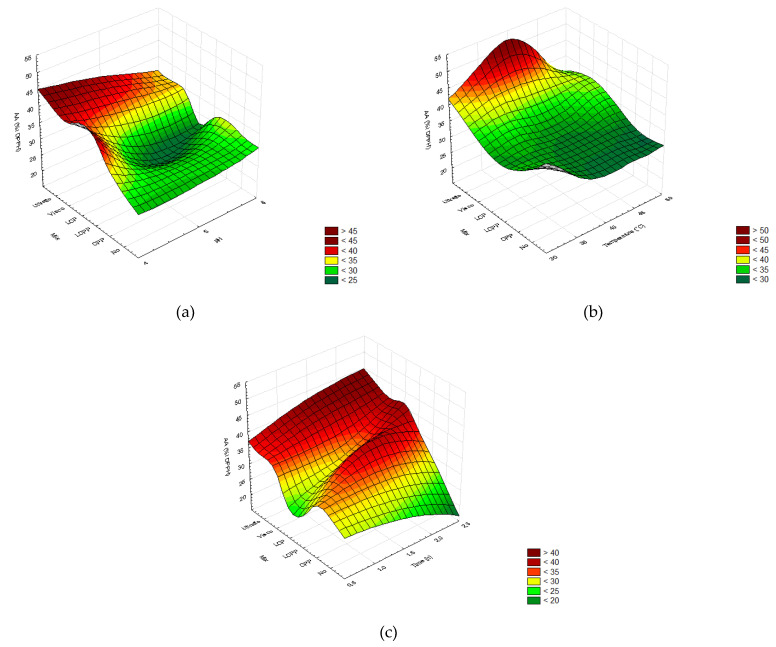
The DPPH-radical scavenging capacity (AA, %I DPPH) in ethanolic extracts plotted as a function of the different enzymatic cocktails. (**a**) pH (4–6), (**b**) temperature (30–50 °C), and (**c**) time (0.5–2.5 h). In order, two commercial cocktails: Ultraflo© (Ultraflo) and Viscozyme© (Visco); three formulated cocktails with laccase, cellulase, and pectinase at proportions of 1:1:1 (LCP), 1:1:2 (LCPP), and 0:1:2 (CPP); and the control without added enzymes (No). The color scale indicates the significance of the analysis of variance for the Taguchi method (*p* > 0.05).

The antioxidant activity (AA) of the extracts subsequently obtained from the hydrolysis performed according to the different DOE showed that all factors had a significant impact (Figure 8). Decreasing pH levels positively influenced the AA of all enzymatic treatments (Figure 8a). The temperature did not impact the AA in the treatment with a formulated enzymatic cocktail but significantly affected when using commercial mixtures (Figure 8b). Finally, the AA increased with time of hydrolysis except in the control treatment (Figure 8c).

**Figure 9:**
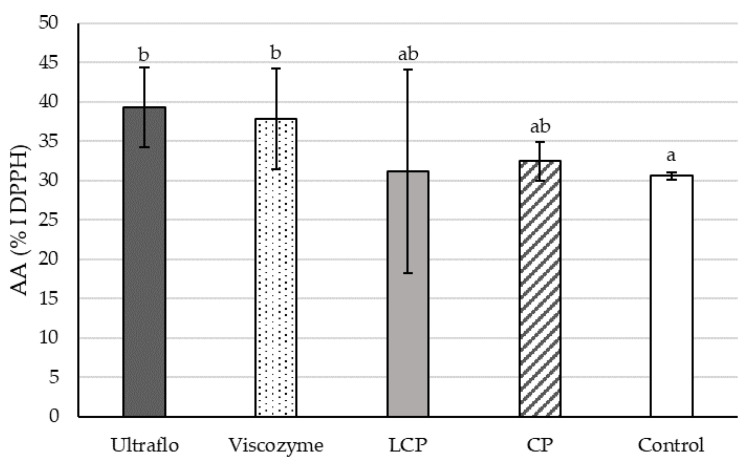
The antioxidant activity (AA) of the ethanolic extracts of *Agave lechuguilla* bagasse estimated through a DPPH radical scavenging assay after enzymatic hydrolysis conducted with two commercial cocktails (Ultraflo© and Viscozyme©), two formulated cocktails with laccase, cellulase, and pectinase at proportions of 1:1:1 (LCP) and 0:1:2 (CP), and the control treatment without enzymes. Different letters indicates statistical significance with the Kruskal–Wallis test (n = 6, *p* < 0.05).

In general, the highest AA was provided by 2.5 h of enzymatic hydrolysis at pH 4 and a temperature up to 40 °C using Ultraflo(c), followed by Viscozyme and LCP (Table A4 and Figure 8). The free radical scavenging capacity of the extracts obtained from processed bagasse reached the highest value using a commercial cocktail from Novozymes^®^, reaching 39.32 ± 5.07 %I (Ultraflo) and 37.86 ± 6.39 %I (Viscozyme). According to the formulated mixture treatment, there was no difference, which resulted in an AA of 31.13 ± 12.93 %I (LCP) and 32.43 ± 2.44 %I (CP). Furthermore, LCP and CP processing did not significantly modulate the AA compared to control conditions (30.59 ± 0.48 %I DPPH) (Figure 9).

#### 2.2.5. Specific Flavonoid Profiles

**Figure 10:**
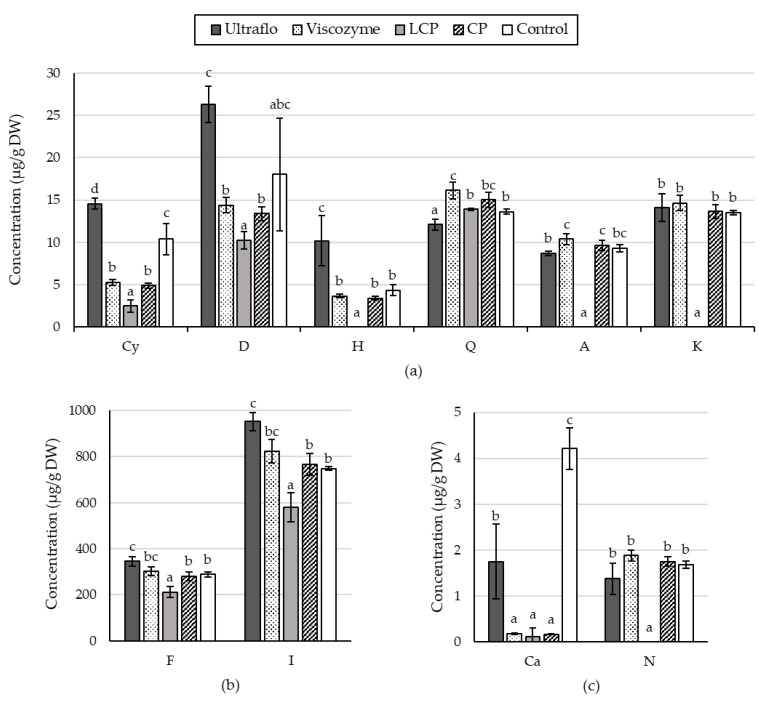
Concentration of flavonoids (µg/g DW) in ethanolic extracts of *Agave lechuguilla* bagasse previously hydrolyzed using two commercial cocktails (Ultraflo© and Viscozyme©), two formulated cocktails with laccase, cellulase and pectinase at proportions of 1:1:1 (LCP), and 0:1:2 (CP), and the control treatment without enzymes. (**a**) cyanidin (Cy), delphinidin (D), hesperidin (H), quercetin (Q), apigenin (A), and kaempferol (K), (**b**) flavanone (F), isorhamnetin (I), and (**c**) catechin (Ca), and naringenin (N). Different letters indicate statistically significant results from the Kruskal–Wallis test (n = 3, *p* < 0.05).

The characterization of the flavonoid profiles by HPLC-UV-MS/MS was assessed on extracts obtained from samples hydrolyzed for 2.5 h at pH 4 with Ultraflo© (26), Viscozyme© (22), LCP (13), CP (14), and control (C1) (Table A1, Table A2, Table A3 and Table A4). The results plotted in Figure 10a show that the concentration of cyanidin in the ethanolic extract was increased using Ultraflo© (14.56 ± 0.64 µg/g DW) and decreased by the other enzymatic cocktails (4.21 ± 1.51 µg/g DW) in comparison to the control (10.39 ± 1.85 µg/g DW). A similar effect was observed on delphinidin variation with a concentration of about 26.33 ± 2.16 µg/g DW of the Ultraflo© treatment, although it was not significant according to the control deviation (18.02 ± 6.68 µg/g DW) (Figure 9). 

Likewise, a higher concentration of hesperidin was obtained with Ultraflo© (10.19 ± 2.99 µg/g DW) and the lowest abundance with LCP (below detection limits) compared to the control (4.34 ± 0.61 µg/g DW). The quercetin concentration was also affected by enzymatic hydrolysis and presented the lowest abundance with the use of Ultraflo© (12.10 ± 0.65 µg/g DW) and the highest value with Viscozyme© (16.14 ± 1.00 µg/g DW) compared to the control (13.61 ± 0.32 µg/g DW) and LCP (13.89 ± 0.65 µg/g DW). The apigenin and kaempferol concentrations fell under the detection limits of the standard curve with the use of the LCP mix, whereas no significant change of their concentration in ethanolic extract was induced by the use of enzymatic digestion compared to the control. 

Apigenin ranged from 8.68 (Ultraflo) to 10.37 (Viscozyme) µg/g DW, and kaempferol, from 13.49 (control) to 14.66 (Viscozyme) µg/g DW. Figure 10b depicts the concentrations of flavanone and isorhamnetin, which showed similar trends among enzymatic treatments. The highest abundances of the two compounds were observed in Ultraflo© treated biomass with 345.43 ± 20.13 and 952.82 ± 39.48 µg/g DW, respectively, although it did not significantly differ from Viscozyme treatment, which provided 30.1.55 ± 18.75 and 823.13 ± 51.18 µg/g DW. 

Furthermore, Viscozyme©, CP, and the control treatment had no significant differences, whereas the lowest values were from the LCP treatment (212.48 ± 22.95 and 581.11 ± 62.34 µg/g DW). Finally, in Figure 10c, the control treatment provided a better concentration of catechin (4.22 ± 0.45 µg/g DW), whereas the naringenin concentration did not differ from that of enzymatic treatment (1.68 ± 0.21 µg/g DW) except for with LCP where it was unquantifiable.

### 2.3. Experimental Validation

The optimal conditions for enzymatic hydrolysis toward phenolic recovery were 2.5 h of incubation at 40 °C in the dark and 180 rpm using the commercial mix Ultraflo© at 2 µL/g of biomass in a phosphate buffer adjusted at pH 4.0. These conditions were reproduced on a larger scale, and the same response variables were evaluated as above. The obtained results shown in Table 2 presented a similar range to the previously obtained during optimization (Table A4). The extraction yield reached 35.53 ± 1.72%, TPC and TFC were about 15.06 ± 1.21 mg GAE/g FW and 7.06 ± 0.98 mg QE/g DW, respectively, and the AA increased to 41.31%I DPPH. In addition, the cyanidin, delphinidin, and hesperidin concentrations significantly increased, and the flavanone, isorhamnetin, and apigenin concentrations decreased due to the enzymatic process (Table 2).

## 3. Discussion

Developing a sustainable biorefinery system for the integral use of *A. lechuguilla* renewable lignocellulosic feedstock was designed. However, optimizing the management and use of the resource is necessary, specifically in early steps of the bioprocess, to ensure the quality of the derived products. The effects of the drying processes and enzymatic hydrolysis on retrieving the phenolic compound as an added-value co-product of the *A. lechuguilla* bagasse is discussed based on the results and proposed from a biorefining perspective.

### 3.1. Drying Process

Drying is crucial in the post-harvest management of plant material to recover phenolic compounds; it allows rapid protection against microbial attacks and chemical alteration due to inner processes, such as oxidation and enzymatic reactions [60,61]. Although the effects of the drying process on health-promoting compounds in foods have been extensively studied, little is known about the impact on agro-industrial waste, such as agave bagasse. Freeze-drying methods are widely accepted as methods that allow for the greater preservation of high-value phytochemicals [62]. 

However, implementing the technology to treat the 150,000 tons of *A. lechuguilla* bagasse produced annually is not viable due to the high operating costs. That is why oven-drying and sun-drying procedures were compared to freeze-drying to study the impact of such processes on the chemical and biological properties of the *A. lechuguilla* agro-waste. Sun-drying is commonly used for medicinal plants, although, because the parameters cannot be controlled, the heterogeneity in the quality of derived products has been highlighted [63]. Oven-drying was chosen as a scalable method allowing fast drying at a controlled temperature [64] set at 40 °C to ensure flavonoid stability [62].

The results showed that oven-dried bagasse presented similar extraction yields to the freeze-dried biomass. The extraction yield from oven-dehydrated and sun-dried material was not significantly different (Figure 1). In contrast to the extraction yields, exposure to light affected the total phenolic content, which was lower for sun-dried and freeze-dried with light. In addition, oven dehydration ensured the same range of TPC as the freeze-drying process (Figure 2). A higher TPC in air-dried than in freeze-dried material was previously attributed to the release of phenolic acid and flavonoids from the plant matrix due to heat [61].

In contrast, freeze-drying in the dark obtained the highest total flavonoid concentration, and no difference could be found among the three other treatments (Figure 2). The effect of light on TFC could not be demonstrated, although the impact of sunlight was inferred by the twice-higher TFC in the extracts obtained from LD compared with S dried bagasse (Figure 2), which concurs with the UV-sensitivity of flavonoids reported in other plant material, such as berries [62] and medicinal herbs [65]. 

This fact is further supported by the verified impact of UV-C on the flavonoid content of *Agave tequilana* extracts. A previous study reported that the 85 °C temperature had more impact on the TFC of *A. tequilana* extracts than the light exposure [49]. In comparison, the lower temperature used for oven dehydration (40 °C) appears to preserve thermal-sensitive flavonoids in the *A. lechuguilla* bagasse. The similar TPC and TFC exhibited by ethanolic extracts of *Agave fourcroydes* oven-dried at a higher temperature (60 °C) [47] support this statement. 

The different drying methods significantly influenced the content of individual flavonoids. Light exposure drastically decreased the anthocyanins content (Table 1), likely due to their particular vulnerability to chemical reactions involving enzymes, light, and oxygen, leading to leakage of components [60,62,64]. In this respect, Leong et al. [52] reported the use of dim light to properly preserve the betacyanins from red-purple pitaya. Similarly, the lowest glycosyl flavonol and flavanone concentrations were found in the sun-dried biomass (Table 1). 

This fact is coherent with the decrease of glycoside flavonoid content observed in full sunlight exposed leaves [65]. In contrast, the constant flavanol and flavanone contents in *A. lechuguilla* bagasse, according to the drying procedure (Table 1), reflected their stability as previously observed in air-dried berries [62]. Finally, the variation of the free-radical scavenging capacity of the extracts was similar to the TPC variation, and the highest inhibition values were obtained from bagasse dried in the dark (Figure 3). 

These results contrast the usual conclusions about lower AA with freeze-drying than oven-drying [62,64]. To sum up, TPC, the individual concentration of glycosyl flavonoids and anthocyanins, and the antioxidant capacity of the extracts were increased in the *A. lechuguilla* bagasse dried in an oven at 40 °C in the dark compared to freeze-drying. Therefore, oven-drying appeared to be the most efficient process to preserve the chemical and biological properties of *A. lechuguilla* bagasse. 

### 3.2. Enzymatic Pretreatment

The recovery of the phytochemicals from plant material is generally limited by physical barriers, e.g., cell walls and membranes. The lignocellulosic matrix of *A. lechuguilla* is composed of 18.3 ± 1.1 to 20.18 ± 1.71% cellulose, 9.7 ± 0.5 to 11.08 ± 0.84% hemicellulose, and 18.9 ± 0.8 to 19.36 ± 0.49% lignin on an oven-dry basis [19,21] depending on the development stage and collection site [66]. The extractive fraction represents from 26.2 to 37.21% [19,21,41], and must contain around 12.28% ± 2.99% of phenolic compounds [38,39], which was corroborated by the current results of extraction yield (38.98 ± 0.64 %DW), and TPC (14.41 ± 1.81 mg GAE/g FW) obtained from oven-dry bagasse (Figure 1 and Figure 2). 

Enzymatic hydrolysis is an efficient pretreatment of the lignocellulosic biomass to enhance the release of bioactive phenolic compounds [57,67]. Uses of lignocellulolytic enzymes and microorganisms for the bioconversion of *A. lechuguilla* bagasse into bioenergy products have been previously studied [18,19,20,21], although the impact of such processes on phytochemicals has not been considered.

Among the enzymes commonly required to degrade the plant matrix and used in both the biorefinery process and active molecule extraction [25,54,55,66,68,69,70,71,72,73], cellulase, pectinase, and laccase were screened to formulate an enzymatic cocktail. The variability of extraction yield explained by the factor pH according to the Taguchi analysis (Figure A2) suggested better activity of the enzymes at pH range from 4 to 5 (Figure A2). In addition, the temperature factor evaluated in the second DOE further induced variability of extraction yields (Figure 4), suggesting an effect of temperature on enzymatic activity. 

This finding is in accordance with the physicochemical characteristic of the purchased enzymes. Cellulase and pectinase acquired from Sigma present optimal activity at pH 5.0 and 37 °C and pH 4.0 at 50 °C, respectively. The fungal laccase provided by the workgroup presents optimal activity at pH 4.0, and is stable at a range of temperatures from 25 to 70 °C with the highest relative activity from 40 to 60 °C [74]. Hence, a pH of around 4 to 5 and temperature of 37–50 °C are recommended for the hydrolysis process using the formulated mix to ensure enzyme activity in the optimal range and facilitate the recovery of phytochemicals from lignocellulosic materials.

Screening of the enzyme amount (5–15 IU) in the formulation revealed that only laccase increased the extraction yield, whereas an increasing concentration of cellulase and pectinase maintained the yield as in the control treatment (Table A4). In addition, the highest TPC and TFC values were obtained with the lowest levels of enzyme concentration (Figure A1 and Figure A2). The Taguchi modeling suggested a positive effect of the pectinase concentration from 5 to 10 IU on the phenolic and flavonoid contents in the extracts (Table A4, Figure A1 and Figure A2). The use of 5 IU (= 2 mg/mL) of cellulase and pectinase previously increased the TFC in *Ginkgo biloba* leave hydrolysates [66]. Similarly, cellulase enhanced the recovery of soluble phenolic compounds from *Psidium guajava* leaves [69].

In contrast, increasing the laccase concentration in the formulation showed a negative impact on TPC and TFC (Figure A1 and Figure A2). Laccases are interesting for waste valorization processes in the biorefinery context because they avoid sugar degradation by acting on the phenolic units of lignin polymers [66]. However, the phenoloxidase activity of the laccases led to the oxidation of phenolic acids [75]. Thereby, the alteration of other phenolic compounds, such as flavonoids, is probable and could explain the present results. In general, oxidation of polyphenols is not recommended because it reduces the antioxidant potential [61]. Low phenol reduction was observed when using bacterial laccases instead of fungal laccases [66]. This option should be considered in the treatment of plant biomass for the procurement of phenolic compounds as added-value co-products. 

Based on these preliminary results, three enzyme mixtures were formulated with laccase, cellulase, and pectinase at the respective proportions of: 1:1:1 (LCP), 1:1:2 (LCPP), and 0:1:2 (CPP). According to the second DOE, the prediction of a positive effect of pectinase amount was refuted since it did not significantly increase the concentrations of phenols and flavonoids in the extracts (Table A4, Figure 4 and Figure 5). On the other hand, a minimum amount of laccase in the formulation improved the extraction yield, as previously obtained in DOE I, compared to treatment without laccase. Surprisingly, phenolics and flavonoids presented the highest concentrations in the presence of laccase in the cocktail (Figure 5 and Figure 6). This could be due to the release of a high free-sugar content allowed by laccase since reducing-sugars are known to interfere with the TPC detection method [76]. 

In addition to the enzyme ratio, the pH, temperature, and treatment time were evaluated in the second DOE because of their effect on enzymatic activity and phenolic compound retrieval. Optimal activity at pH 4, suggested in the first DOE (Figure A1 and Figure A2), was confirmed by the results of DOE II. Therefore, the highest yield (Figure 4a), TPC (Figure 5a), and TFC (Figure 6a) were obtained for pH 4 (Figure 4a, Figure 5a, and Figure 6a). Likewise, the total phenolic and flavonoid recovery were improved with incubation time (Figure 5c and Figure 6c), whereas the tested temperature range had no significant impact (Figure 5b and Figure 6b). The PCA confirmed this result (Figure 7), which concluded that enzyme mix composition was the main factor influencing the phenolic and flavonoid recovery from *A. lechuguilla* hydrolyzed bagasse.

The formulated mixture’s efficiency toward flavonoid extraction was compared to a commercial enzymatic cocktail. A third DOE was performed to evaluate the impact of pH, temperature, and incubation time on the hydrolysis of *A. lechuguilla* bagasse with 2 mg/mL of Ultraflo© and Viscozyme© purchased from Novozymes^®^. The Ultraflo© cocktail is characterized by cellulase and xylanase activity. Viscozyme© contains a wide range of carbohydrases, including arabanase, cellulase, β-glucanase, hemicellulase, and xylanase. Optimum conditions for the enzymatic activity of Ultraflo© are pH 6.0 and temperature of 50–60 °C, and for Viscozyme© are pH 3.3–5.5 and temperature 40–50 °C. 

In the present study, the pH tested from 4 to 6 did not impact the TPC and TFC in the extracts, whereas reducing the temperature from 50 to 30 °C, and increasing the incubation time from 0.5 to 2.5 h showed a positive effect on the TPC and TFC (Figure A4 and Figure 5 and Figure 6). Similarly, Viscozyme© used at 0.2% was previously found to allow higher phenolic recovery from *Brassica oleracea* leaves in comparison with control, and the authors reported no effect of pH (3–6) and temperature range (30–50 °C) [58]. Likewise, increasing the time enhanced the phenolic recovery from *Rosmarinus officinalis* leaves hydrolyzed with Viscozyme©, although longer than a 3 h incubation time was found to have a negative effect [76]. In contrast, time does not affect the phenolic recovery using Ultraflo© at pH 5.0 and 50 °C for 6 h [77] and pH 4.0 and 40 °C for 12 h [78]. 

In comparison to the formulated enzyme mixtures, the two commercial cocktails were more efficient for the recovery of phenolic and flavonoid content, and the highest TPC and TFC were obtained with Ultraflo© compared to Viscozyme© (Figure 5, Figure 6 and Figure 7). Ultraflo© and Viscozyme© enhance the TPC in *Opuntia humifusa* hydrolysate [72], and the Viscozyme© efficiency was confirmed for the recovery of specific flavonoids from *Opuntia ficus-indica* [67], which could be attributed to the presence of xylanases in both cocktails, which allows for a better degradation of the hemicellulose of *A. lechuguilla* composed at 63% of xylose [21]. Thus, xylanase should be considered in future mix formulations, such as those proposed by Wang et al. [69]. In addition, if Viscozyme© is preferred for cactus mucilage digestion to retrieve phenolics [67,77], the Ultraflo© appeared to be more specific and efficient to release phenolic compounds from the lignocellulosic matrix of *A. lechuguilla*. 

In general, enzymatic hydrolysis enhanced the TPC and TFC, which increased the radical scavenging capacity of the extracts compared to the control treatment (Figure 8 and Figure 9). Likewise, enzymatic-assisted extraction improves the DPPH scavenging capacity of *R. officinalis* extracts due to a higher TPC than conventional extraction methods [76], and enzymatic pretreatment of *P. guajava* leaves enhances the DPPH values by 2.3 fold [69]. Both the highest and lowest DPPH radical scavenging values were observed with laccase depending on the pH, temperature, and hydrolysis time (Figure 8). 

The release of reducing sugar and phenolic acids by the action of laccase likely participates in the AA [72], explaining the large deviation. Lower variation and 1.5-fold increased AA results were obtained from *A. lechuguilla* bagasse hydrolyzed by Ultraflo© and Viscozyme© for 2.5 h, at 40 °C and pH 4 (Figure 8). Similarly, the DPPH radical scavenging capacity of *O. humifusa* hydrolysates increases using Ultraflo© and Viscozyme© [77]. The use of enzyme-assisted extraction for the obtention of bioactive flavonoids from diverse biomass is globally accepted. However, it has been reported that enzymes significantly modulate the flavonoid profile of the extracts, which impacts the antioxidant capacity [67,79]. In addition, the anti-cancer activity of *A. lechuguilla* decreases in acid hydrolyzed extracts [32]. Hence, a specific flavonoids profile must be characterized to obtain further conclusions regarding the optimal enzymatic pretreatment. 

In this study, 10 flavonoids previously described in *A. lechuguilla*-untreated material were quantified in the extract obtained after enzymatic hydrolysis executed for 2.5 h at pH 4 with Ultraflo© (26), Viscozyme© (22), LCP (13), CP (14), and control (C1). The alteration of flavonoid profile by laccase, as previously suggested, was confirmed by the reduced concentration of all quantified flavonoids, except quercetin, compared to the other treatments (Figure 10). In particular, cyanidin decreased by 43.5%, delphinidin by 21.2%, and flavanone by 1.5%. In addition, drastic decreases in the kaempferol, apigenin, naringenin, and hesperidin contents were observed, which went below the detection limits (Figure 10).

In contrast, Ultraflo© increased the content of hesperidin by 2.5 fold, cyanidin by 1.5 fold, delphinidin by 1.4 fold, isorhamnetin by 1.3 fold, and flavanone by 1.2 fold in the extracts compared to the control, and the obtained concentrations were higher when compared with Viscozyme© and CP. The quercetin concentration in the extract was reduced by using Ultraflo© and increased by the use of Viscozyme©. Furthermore, the apigenin, kaempferol, and naringenin concentrations remained at the same concentration as untreated bagasse (Figure 10). 

The anthocyanins, flavanone, isorhamnetin, quercetin, and kaempferol quantified fractions contain glycoside derivatives [40,41]. Hence, a decrease of these fractions agrees with the previously reported bioconversion of phenolic glycosides into their aglycones caused by the use of Viscozyme©. Wang et al. [69] described a loss of glycosyl quercetin derivatives, and aglycone quercetin and kaempferol gain. Similarly, Antunes-Ricardo et al. [67] demonstrated the breakdown of the sugar moiety from original triglycosylated forms of isorhamnetin and quercetin. Kim et al. [77] reported a decrease in the quercitrin concentration and an increase in the quercetin and isorhamnetin contents after enzymatic hydrolysis. 

In contrast, the Ultraflo© and CP mix likely preserved glycoside forms and even enhanced their recovery in the case of Ultraflo©. The efficiency of cellulase and pectinase for recovering flavonol aglycones, such as quercetin, kaempferol, and isorhamnetin, from *G. biloba* leaves has been demonstrated [70]. Likewise, using a mix prepared with pectinase and cellulase (1:2, *w*/*w*), it was possible to extract 16 flavonoids, including glycoside conjugates with higher yields than without enzymatic pretreatment [73]. Transglycosylation activity of the enzymes can explain the lowest loss of glycosyl flavonoids in the Ultraflo© and CP treatment [70]. 

On another hand, pH and temperature could affect the flavonoid profile not only in the hydrolysis process but also in the extraction process. Tran et al. [56] demonstrated that temperature and time particularly impact the extraction of isoflavones and reported that pH may affected the isoflavone structure. These modifications on the flavonoid profile are relevant due to the well-studied structure–activity relationship. The release of aglycone flavonols is particularly interesting for pharmaceutical applications [58,67,69,79]. At the same time, enhanced glycosyl flavonoids, particularly the anthocyanidin content, suggest applications in cosmetics and nutraceuticals [80].

Therefore, Ultraflo© at pH 4.0, at 40 °C, and for 2.5 h were considered in this study to be the best hydrolysis parameters to enhance bioactive flavonoid recovery from *A. lechuguilla* bagasse. Experimental validation was conducted, and the results confirmed the increase of the extraction yield, total phenolic recovery, DPPH radical scavenging, and anthocyanidin concentrations (Table 2). The highest concentration of anthocyanins supports the use of enzymatic treatment for the procurement of high added-value co-products. Thus, despite the fact that the high cost of enzymes is usually considered a limitation for scaling-up [53], a techno-economic evaluation should be performed to reconsider the enzyme-assisted extraction within biorefinery schemes. 

## 4. Materials and Methods

### 4.1. Plant Feedstock

*Agave lechuguilla* bagasse was obtained in August 2018 from the Ejido Cosme, Ramos Arizpe, Coahuila (GPS: 25°52′03.6″ N; 101°19′51.1″ W). Gatherers collected stem leaves according to Mexico’s Official Standards for central stem harvesting and land shifts (NOM-008-SEMARNAT-1996) [13] and recovered the fiber through a mechanical process that generates pulpous residue, which was immediately harvested and cryopreserved at −80 °C.

### 4.2. Drying Procedure

Four drying methods were applied, each one on 500 g of fresh bagasse to evaluate the effect of light and temperature on flavonoid content. As the most efficient laboratory-scale method, freeze-drying was performed for 48 h at −49 °C under a vacuum (Labconco equipment, Kansas City, MO, USA) in the dark (LD) and artificial light (LL). In addition, scalable alternative methods were tested, sun drying (S) (as this can occur at the harvest sites) (35 ± 5 °C), and dehydration at 40 °C in the dark (D) (Koleff-KL10 tray convection oven, Queretaro, QRO, Mexico), both for about 24 h, until reaching a <10% (*w*/*w*) moisture content. 

The moisture content was estimated by the weight difference, calculated by burning 500 mg of dehydrated material at 120 °C for 15 min (Thermobalance MB45, OHAUS, Mexico City, Mexico). The dry material was milled into 2 mm particle size powder (Retsch-SM100 Industrial Mill, Retsch Co., Haan, Germany) and stored at room temperature, preventing exposure to light, oxygen, and moisture until the phytochemical extractions were performed.

### 4.3. Enzymatic Hydrolysis

#### 4.3.1. Optimization Method

The design of experiments (DOE) based on the Taguchi method was used to optimize the enzymatic hydrolysis process. The parameters considered for the enzymatic treatment were the enzymatic mix composition, pH, temperature, and incubation time.

According to the biochemical composition of the *A. lechuguilla lignocellulosic* biomass, cellulase (C, endo-1,4-β-D-glycosidase activity, from *Aspergillus niger,* Sigma-Aldrich, Mexico) and pectinase (P, polygalacturonase activity, from *A. niger,* Sigma-Aldrich, Mexico) as cellulolytic and hemicellulolytic enzymes were required. In addition, to target lignin specifically, the use of laccase (L) (from *Pycnoporus sanguineus* CS43, Grupo SAB, ITESM, Mexico) was evaluated. A first DOE aimed to establish the proportion of the three enzymes in the mix. The minimum amount of each enzyme was set at 5 IU, and the maximum was set at 15 IU. 

The pH was considered the fourth parameter due to its critical influence on enzymatic activity, and the tested pH ranged from 4 to 6. pH conditions (4, 5, and 6) were also tested as controls, without enzyme addition. The pH of the phosphate buffer, previously prepared at 0.1 M, was adjusted by adding concentrated citric acid (10 M). Considering the four variables with three values each, a Taguchi L9 orthogonal array was applied (Table A1). The enzymatic digestion was carried out with 300 mg of dry matter in a final volume of 10 mL and incubated in the dark for 1 h at 40 °C with shaking at 180 rpm. The enzymatic reaction was stopped by freezing at −80 °C before drying at −49 °C and 0.080 Pa in the dark. 

After that, a second Taguchi L9 DOE was assessed to evaluate the efficiency of three enzymatic mixes, L:C:P: (1) 0:5:10 IU, (2) 5:5:10 IU, and (3) 5:5:5 IU; under different pH (4–6), temperature (30–50 °C) and incubation time (0.5–2.5 h) conditions (Table A2). 

A third DOE was conducted using 2 mg/mL of Viscozyme© and Ultraflo© purchased from Novozymes^®^ to compare the formulated mix with commercial cocktails. The effect of pH, temperature, and incubation time was evaluated at the same value range as in the second DOE. The four factors with two values were tested through a Taguchi L8 matrix of experiments (Table A3).

#### 4.3.2. Experimental Validation

The optimal hydrolysis parameters were verified by reproducing the enzymatic digestion of 50 g of oven-dried *A. lechuguilla* bagasse. The dry powder was placed in a 1 L flask with 500 mL of phosphate buffer (0.1 M) at pH 4. Incubation occurred in the dark at 40 °C, 180 rpm for 2.5 h. The enzymatic mix Ultraflo (liquid) was added at a ratio of 3.31 µL/g of biomass.

The whole flask content was transferred to layered trays and dried at 40 °C in the dark for 24 h. The dried hydrolyzed material was stored at room temperature, preventing light, oxygen, and moisture exposure until the phytochemical extraction. 

### 4.4. Ultrasound-Assisted Extraction

The phytochemicals were obtained by Ultrasound-Assisted Extraction (UAE) from the dried and powdered residue homogenized with ethanol/water (70/30, *v*/*v*) using a proportion of 1/10 (*w*/*v*). The UAE was performed three consecutive times for 45 min, 80.0 Hz, and 40 °C, collecting and changing the solvent between each incubation. The supernatants were pooled, filtered at 0.22 µm (Whatman™ Uniflow™ Syringe Filters), and concentrated at 60 °C through vacuum rotary evaporation (IKA, Wilmington, NC, USA). The extracted phytochemicals were solubilized again in distilled water, frozen at −80 °C, and freeze-dried (−49 °C, 0.080 Pa) (Labconco Equipment) for 24 to 48 h, depending on the obtained volume. Finally, the extraction yields were determined for each triplicate of ethanolic extracts (EtOH) and reported as the dry basis or fresh weight according to the moisture content of the initial biomass.

### 4.5. Phytochemicals Profiling

#### 4.5.1. Preparation of Extracts

For the total polyphenol content (TPC), total flavonoid content (TFC) determination, and free radical scavenging assay, 10 mg of each triplicate recovered of ethanolic extracts was solubilized in distilled water to reach a 10 mg/mL concentration. Dilutions were prepared at 2, 1, and 0.5 mg/mL. 

For HPLC-UV-MS analyses, ethanolic and methanolic extracts were dissolved at 1 mg/mL in methanol/water (50/50, *v*/*v*), HPLC grade solvents (Fermont, Mexico, www.pqm.com.mx, accessed on 3 November 2021) and filtered thought Whatman 0.45 µm nylon filters.

#### 4.5.2. Total Phenolic and Flavonoid Contents

The extracts’ total phenolic content (TPC) was estimated according to the adapted microplate protocol from Singleton and Rossi [81]. Briefly, 20 µL of extract and negative and positive controls were placed in a 96-well flat-bottom plate, and 10 µL of Folin-Ciocalteu reagent (Sigma-Aldrich, Mexico) was added, followed by 40 µL of Na_2_CO_3_ at 200 g/L and 130 µL of distilled water. After incubating 30 min at 40 °C in the dark, the optical density was read at 735 nm by the Epoch microplate reader (Biotek Instruments, Winooski, VT, USA). The phenol concentrations were obtained in milligrams of gallic acid equivalent (GAE) by reference to the standard curve (y = 0.0057x +0.0023, R² = 0.9997) and reported per gram of fresh weight (g FW) considering the moisture content.

The extract’s total flavonoid content (TFC) was determined by applying the aluminum chloride method [82] adapted to the microplate. In brief, 20 µL of extracts and negative and positive controls, 7.5 µL of NaNO_2_ at 5%, 30 µL of 2.5% AlCl_3_ (Jalmek, Mexico www.jalmek.com) solution, 50 µL of NaOH at 1 M, and 50 µL of distilled water were deposited in that order into the 96-well flat-bottom plate with 5 min homogenization between each addition. The DO was measured at 500 nm by the Epoch microplate reader (Biotek Instruments). The flavonoid concentrations were estimated in milligrams quercetin equivalent by reference to the standard curve (y = 0.0009x +0.0451, R² = 0.9928) and reported per gram of fresh weight (mg QE/g FW).

#### 4.5.3. HPLC-UV-MS/MS

The quantification of apigenin, catechin, cyanidin, delphinidin, flavanone, hesperidin, and isorhamnetin in the ethanolic extracts was achieved using flavonoid analytical standards (Sigma-Aldrich) as reported by Morreeuw et al. [40].

Reverse phase high-performance liquid chromatography (RP-HPLC) analysis was performed according to Mendez-Flores et al. [83] on a Varian HPLC system (Agilent Technologies, Santa Clara, CA, USA), including an autosampler (Varian ProStar 410, Agilent Technologies), a ternary pump (Varian ProStar 230I), and a photodiode array detector (PDA, Varian ProStar 330, USA). Briefly, samples (5 µL) were injected onto a Denali^®^ C18 column (150 mm × 2.1 mm, 3 µm, Grace, Williamsburg, MI, USA) maintained at 30 °C. The mobile phase consisted of formic acid (0.2 %, *v*/*v*; solvent A) and acetonitrile (solvent B). 

The following gradient was applied: initial, 3% B; 0–5 min, 9% B linear; 5–15 min, 16% B linear; and 15–45 min, 50% B linear. The column was then washed and reconditioned. The detection of released compounds was performed through the PDA detector at 280 and 360 nm. The UV spectra were analyzed using Chromatography Workstation Star Toolbar (version 6.30) software from Agilent for the Varian equipment.

### 4.6. Free-radical Scavenging Capacity

The antioxidant potential of the extracts was estimated by performing a DPPH-radical (2,2-diphenil-1-picrihydrazil, Sigma-Aldrich, Mexico) scavenging assay as described by Brand-Williams et al. [84] and adapted to the microplate. Ethanol and distilled water were used as a negative control. The charged microplates were incubated for 30 min in the dark at room temperature (≤25 °C). The absorbance was measured at 540 nm in an Epoch microplate reader (Biotek Instruments). The antioxidant activity (AA) of the extracts was reported as the percentage of inhibition of the DPPH reactive (% I DPPH = [(Abs control − Abs extract)/Abs control) × 100]).

### 4.7. Statistical Analysis

To compare the drying treatments, we analyzed the technical and extractive replicates for the global yield, TPC, TFC, quantitative HPLC-UV, and AA data with the Shapiro–Wilk test for normality and the Bartlett test for homoscedasticity. When the two main statistical assumptions of analysis of variance (ANOVA) were verified, ANOVA was run, followed by a Tukey HSD test to determine significant pairwise differences. When normality was not respected, the Kruskal–Wallis test was applied as a non-parametric alternative to ANOVA. All statistical tests were performed with an alpha of 0.05 in the R programing language (R Core Team, 2020) version 4.1.0. 

The design of the experiment matrix was obtained, and response variables (TPC, TFC, flavonoid profiles, and AA) were preliminarily analyzed and visualized with JMP software (version 5). Deep screening of the effect of each tested factor on response variable was assessed within each DOE and merging all the data of the three DOE to establish the optimal pretreatment conditions. Principal component analysis (PCA) and multifactorial analysis were obtained using R (R Core Team, 2020) version 4.1.0. The highest signal-to-noise (S/N) ratio of the levels of the considered factors in the DOE indicates an optimal level. The level of the response variables and ANOVA for Taguchi method based on the S/N ratio results were plotted using Statistica software (version 8).

## 5. Conclusions

As a new raw material that is already valuable for bioenergy production, the *A. lechuguilla* contains specialized metabolites, such as bioactive flavonoids. In this perspective, the post-harvest management and pretreatment of the biomass were optimized, considering the future application at industrial levels. Therefore, the oven-drying process is proposed as a scalable alternative to freeze-drying. This process preserved the flavonoid content of the *A. lechuguilla* bagasse, and the antioxidant capacity was observed. Pretreatment of the lignocellulosic biomass by enzymatic hydrolysis was evaluated toward specific flavonoid recovery.

The results indicate that enzymatic cocktails should be carefully selected according to the bioactive compounds aimed to be extracted. The optimal conditions to enhance flavonoid glycoside recovery, specifically the anthocyanidin content, were found to be the commercial mix Ultraflo© for 2.5 h at 40 °C and pH 4. The potential applications of the active flavonoids in pharmaceutical, cosmetic, and nutraceutical industries suggest the high value of the derived products. Hydrolysis of the polymeric matrix should be confirmed by further analysis regarding the fiber and sugar contents in the *A. lechuguilla* hydrolysates. Finally, this study promotes the integration of phenolic compound extraction in the biorefining of *A. lechuguilla* agro-residue as a step forward to process sustainability and circularity.

## Figures and Tables

**Table 1:** Concentration of flavonoid ^‡^ in micrograms per gram DW (µg/g DW) obtained by HPLC-UV quantitative analysis of ethanolic extract of *Agave lechuguilla* bagasse dried under different conditions ^∫^.

		LD			LL			D			S	
	Means	SD	*	Means	SD	*	Means	SD	*	Means	SD	*
F	224.780	9.795	c	179.290	1.373	ab	194.120	5.180	bc	151.925	4.205	a
I	614.320	12.470	b	490.720	6.404	a	864.350	7.700	c	458.956	2.039	a
Ca	4.970	2.260	a	2.110	1.320	a	3.340	1.600	a	1.814	0.071	a
Cy	3.530	0.220	b	0.000	0.000	a	6.590	1.190	c	0.000	0.000	a
D	11.420	0.320	b	0.000	0.000	a	10.880	0.990	b	0.000	0.000	a
H	4.050	0.290	b	1.830	0.800	a	3.720	0.200	b	1.388	0.477	a
Q	13.730	0.250	c	12.280	0.680	b	13.620	0.380	bc	10.746	0.652	a
A	7.850	0.840	a	8.640	0.490	a	8.240	0.600	a	7.813	0.430	a
K	12.770	0.430	ab	13.170	0.250	b	12.960	0.310	ab	12.122	0.375	a
N	1.430	0.150	a	1.570	0.090	a	1.500	0.110	a	1.239	0.189	a

* The given letters “a”, “b”, and “ab” indicate a significant difference in flavonoid ^‡^ concentrations as a function of the drying process ^∫^. Kruskal–Wallis analysis of variance (n = 3, *p* < 0.05). SD, standard deviation. ^‡^ F, flavanone; I, isorhamnetin; Ca, catechin; Cy, cyanidin; D, delphinidin; H, hesperidin; Q, quercetin; A, apigenin; K, kaempferol; and N, naringenin. ^∫^ LD, freeze-dried in the dark; LL, exposed to light; D, oven-dehydrated; and S, sun-dried.

**Table 2:** The extraction yield, total phenolic content (TPC), total flavonoid content (TFC), antioxidant activity (AA), and specific flavonoid concentrations in ethanolic extracts obtained from unprocessed and processed bagasse of *Agave lechuguilla*.

	Unprocessed		Processed		
	Mean	SD	Mean	SD	
Yield (% DW)	31.65 ± 1.65		35.53 ± 1.72		*
TPC (mg GAE/g DW)	12.81 ± 0.73		15.06 ± 1.21		*
TFC (mg QE/g DW)	6.41 ± 1.02		7.06 ± 0.98		
AA (%I DPPH)	35.59 ± 2.68		41.31 ± 2.14		*
**Flavonoids (µg/g DW)**					
Flavanone	194.120 ± 5.180		147.238 ± 5.390		*
Isorhamnetin	864.350 ± 7.700		752.796 ± 2.908		*
Catechin	3.340 ± 1.600		4.377 ± 0.500		
Cyanidin	6.590 ± 1.190		10.481 ± 2.605		*
Delphinidin	10.880 ± 0.990		21.549 ± 3.794		*
Hesperidin	3.720 ± 0.200		4.690 ± 0.187		*
Quercetin	13.620 ± 0.380		13.481 ± 0.318		
Apigenin	8.240 ± 0.600		3.252 ± 0.608		*
Kaempferol	12.960 ± 0.310		13.482 ± 0.311		
Naringenin	1.500 ± 0.110		1.682 ± 0.111		

* Indicates significant difference between treatments (ANOVA, n = 3, *p* < 0.05).

## Appendix A

### Appendix A.1. Method

**Table A1:** Randomly generated Taguchi L9 orthogonal array for the experiment design with four variables and three factors: pH and concentrations of laccase, cellulase, and pectinase in IU.

Taguchi L9	pH	Laccase (UI)	Cellulase (UI)	Pectinase (UI)	Pattern
1	4	5	5	5	−−−−
2	4	10	10	10	−000
3	4	15	15	15	−+++
4	5	5	10	15	0−0+
5	5	10	15	5	00+−
6	5	15	5	10	0+−0
7	6	5	15	10	+−+0
8	6	10	5	15	+0−+
9	6	15	10	5	++0−

**Table A2:** Randomly generated Taguchi L9 orthogonal array for the experiment design with four variables and three factors: pH, the proportion of laccase, cellulase, and pectinase in the mix, temperature, and incubation time.

Taguchi L9	pH	Ratio L:C:P (UI)	Temp (°C)	Time (h)	Pattern
11	4	0:5:10	30	0.5	−−−−
12	4	5:5:10	40	1.5	−000
13	4	5:5:5	50	2.5	−+++
14	5	0:5:10	40	2.5	0−0+
15	5	5:5:10	50	0.5	00+−
16	5	5:5:5	30	1.5	0+−0
17	6	0:5:10	50	1.5	+−+0
18	6	5:5:10	30	2.5	+0−+
19	6	5:5:5	40	0.5	++0−

**Table A3:** Randomly generated Taguchi L8 orthogonal array for the experiment design with four variables and two factors: commercial mix, pH, temperature, and incubation time.

Taguchi L8	Mix	pH	Temp (°C)	Time (h)	Pattern
21	Viscozyme	4	30	0.5	−−−−
22	Viscozyme	4	50	2.5	−−++
23	Viscozyme	6	50	0.5	−+−+
24	Viscozyme	6	30	2.5	−++−
25	Ultraflo	4	50	0.5	+−−+
26	Ultraflo	4	30	2.5	+−+−
27	Ultraflo	6	30	0.5	++−−
28	Ultraflo	6	50	2.5	++++

### Appendix A.2. Results

**Table A4:** The extraction yields, total phenolic content (TPC), total flavonoid content (TFC), and free-radical scavenging capacity (AA) in response to the different enzymatic digestion treatments applied in the three designs of the experiment (DOE I, II, and III).

	Yield (%FW)		TPC (mg GAE/g FW)		TFC (mg QE/g FW)		AA (%I DPPH)	
Controls								
C1	18.753 ± 2.416	ab	15.518 ± 0.302	ad	4.811 ± 0.094	bcehij	31.031 ± 1.384	cd
C2	18.662 ± 1.116	ab	13.099 ± 1.217	ab	3.668 ± 0.341	ae	30.082 ± 1.108	bcd
C3	18.327 ± 1.015	ab	12.840 ± 1.570	ab	3.788 ± 0.463	ae	30.655 ± 1.458	bcd
DOE I								
1	22.202 ± 2.028	ace	12.842 ± 0.394	ab	4.026 ± 0.123	acef	NA	
2	22.652 ± 2.701	ace	12.251 ± 1.032	ab	3.693 ± 0.311	ae	NA	
3	27.763 ± 2.985	e	11.675 ± 0.908	ab	3.339 ± 0.260	acef	NA	
4	26.271 ± 1.878	de	12.419 ± 1.540	ab	3.962 ± 0.491	ae	NA	
5	21.060 ± 0.824	acd	12.301 ± 0.606	ab	4.465 ± 0.220	bceh	NA	
6	23.290 ± 1.245	ace	12.345 ± 1.270	ab	3.259 ± 0.335	acef	NA	
7	24.509 ± 2.584	bce	12.885 ± 0.073	ab	3.519 ± 0.159	acd	NA	
8	25.451 ± 3.926	ce	11.908 ± 0.831	ab	3.078 ± 0.215	ab	NA	
9	22.721 ± 1.759	ace	10.939 ± 0.679	ab	2.623 ± 0.163	a	NA	
DOE II								
11	19.341 ± 2.734	ac	13.865 ± 1.465	abc	5.269 ± 0.557	dehij	34.061 ± 4.633	cde
12	20.419 ± 3.881	acd	16.166 ± 1.809	ad	5.412 ± 0.181	ehij	44.372 ± 4.850	ghi
13	21.792 ± 0.548	ace	20.256 ± 2.870	d	5.834 ± 0.827	fghk	45.530 ± 2.607	h
14	21.782 ± 1.046	ace	13.215 ± 2.286	abc	4.625 ± 0.800	bcehi	29.619 ± 1.592	bcd
15	21.516 ± 3.371	ace	12.335 ± 0.363	ab	4.687 ± 0.138	bcehi	23.176 ± 0.858	ab
16	21.334 ± 1.596	ace	12.899 ± 0.631	ab	4.946 ± 0.277	cehij	27.327 ± 1.263	ac
17	21.284 ± 1.272	ace	11.283 ± 2.014	a	3.986 ± 0.585	ae	33.602 ± 0.304	cde
18	16.856 ± 1.398	a	14.716 ± 3.163	abc	4.934 ± 0.572	cehij	34.434 ± 1.150	cdef
19	22.779 ± 0.908	ace	11.893 ± 0.743	ab	4.358 ± 0.218	aceh	20.527 ± 1.540	a
DOE III								
21	20.027 ± 1.737	acd	15.696 ± 1.397	ad	5.933 ± 0.528	hk	36.931 ± 2.278	dg
22	23.305 ± 3.603	ace	15.143 ± 1.341	ad	5.833 ± 0.517	fghk	41.797 ± 3.616	fgh
23	22.911 ± 1.380	ace	11.640 ± 0.922	ab	4.093 ± 0.324	aceg	29.236 ± 3.471	bc
24	22.991 ± 0.507	ace	18.635 ± 3.810	cd	7.380 ± 1.509	k	43.496 ± 1.789	gh
25	18.892 ± 1.566	ac	15.517 ± 1.901	ad	6.129 ± 0.780	hk	32.305 ± 1.057	cd
26	20.394 ± 0.906	acd	15.167 ± 1.586	ad	6.361 ± 0.057	jk	40.761 ± 3.224	gh
27	19.747 ± 0.632	acd	15.790 ± 0.141	bd	5.919 ± 0.570	ghk	39.846 ± 2.517	egh
28	21.568 ± 1.648	ace	17.132 ± 3.832	ad	6.624 ± 1.482	ik	44.374 ± 0.905	egh

The means and SD based on triplicate analyses of the results obtained for each response variable. Different letters indicate pairwise difference obtained from the Tukey HSD test applied after the multifactorial ANOVA (n = 6; *p* < 0.05).
